# Advancing inclusive research calculator for oncology disease areas: A resource to support the development of enrollment targets in diversity action plans for industry sponsors

**DOI:** 10.1371/journal.pone.0315283

**Published:** 2024-12-19

**Authors:** David J. Press, Spencer L. James, Bruno Jolain, Nicole Richie

**Affiliations:** 1 A Member of the Roche Group, Genentech, South San Francisco, California, United States of America; 2 Department of Population Health Sciences, Duke University School of Medicine, Durham, North Carolina, United States of America; 3 F. Hoffman-La Roche Ltd., Basel, Switzerland; The University of Sydney, AUSTRALIA

## Abstract

**Background:**

The Food and Drug Omnibus Report Act, signed into law in 2022, requires industry sponsors to include diversity action plans in clinical study protocols. Defining reliable methodology for measures and benchmarks is critical to ensuring adequate and consistent representation of historically underrepresented patient populations in clinical trials.

**Methods:**

We provide an Advancing Inclusive Research (AIR) Calculator, summary tables, and data query bank to support target setting for the development of diversity action plans and to take steps toward defining enrollment standards. The AIR Calculator uses data from the US Cancer Statistics database, which covers 100% of the US population. The database provides descriptive statistics for people diagnosed with 26 different cancers from 2015–2019 by cancer site, age at diagnosis, sex, and race and ethnicity, all stratified by stage at diagnosis (early, de novo metastatic, and combined). Descriptive characteristics include frequency counts, age-adjusted incidence rates, incidence rate ratios, and 95% CIs. Robustness test results are available in the data query bank by year of diagnosis.

**Results:**

This resource offers insights into distributions of cancer in the US. The AIR Calculator allows users to calculate representative clinical study distributions based on the sponsor-designated study size.

**Discussion:**

The AIR Calculator serves as a valuable resource for planning of clinical studies, but additional data analyses are necessary for a comprehensive understanding at the study level. Comprehensive data collection and alignment across industry are essential to ensure consistent, accurate, and transparent benchmarks in historically underrepresented patient populations and to track progress toward the goal of improving their representation in clinical research.

## Introduction

On December 29, 2022, the Food and Drug Omnibus Report Act was signed into law by the US president. The act includes provisions that will go into effect in 2025 requiring industry sponsors to include diversity action plans when submitting protocols for pivotal/Phase 3 clinical studies to the US Food and Drug Administration (FDA) [1]. In practice, the FDA is already enforcing the requirement to improve representation of racial and ethnic groups in clinical studies in advance of the mandatory timeline, as evidenced by the increasing number of post marketing commitments and requirements focused on evidence generation in underrepresented patient populations for newly approved medicines [2]. These changes are part of the FDA’s emerging guidance for industry sponsors to ensure that clinical study populations are representative of the patient populations likely to use the medical product in the future, with a focus on historically underrepresented populations such as women, racial and ethnic minority groups, and elderly individuals [3, 4].

Defining and consistently using accurate benchmarks and standards to improve representation of historically underrepresented patient populations are crucial for ensuring transparency and alignment across the pharmaceutical industry. The Advancing Inclusive Research (AIR) Calculator for cancer areas is specifically designed to support the development of enrollment targets in diversity action plans. In addition to its calculator functionality, the AIR Calculator includes summary tables and a data query bank. The AIR Calculator provides representative distributions of clinical study participant numbers based on the distribution of disease in the US population by age at diagnosis, sex, and race and ethnicity, all stratified by stage at diagnosis.

## Materials and methods

### Data sources

Cases of incident cancer are registered in every US state and cover the entire US population as a feature of federal and state laws mandating the registration of reportable diseases, including neoplasms [5]. The AIR Calculator uses information from US Cancer Statistics (USCS) data, which combine cancer registry data from the Centers for Disease Control and Prevention’s (CDC’s) National Program of Cancer Registries and the National Cancer Institute’s (NCI’s) Surveillance, Epidemiology, and End Results (SEER) program [6]. This database contains deidentified publicly available data and did not require institutional review board approval or patient written consent. This dataset includes cancer incidence data from central cancer registries in 50 states (National Program of Cancer Registries in 46 states and the District of Columbia and SEER in 4 states) [7]. Data on all new diagnoses of cancer in patient records at medical facilities (eg, hospitals, physicians’ offices, therapeutic radiation facilities, freestanding surgical centers, and pathology laboratories) are reported to central cancer registries, which collate these data and use state vital records to collect information on any cancer-related deaths that were not reported as cases. The central cancer registries use uniform data items and codes as documented by the North American Association of Central Cancer Registries. These data are submitted annually to the CDC and NCI and combined into one dataset [8]. Cancer registries demonstrate that data are of high quality by meeting US Cancer Statistics publication criteria [6]; during 2015–2019, data from both registries, covering 100% of the US population, met the publication criteria.

The AIR Calculator includes new cases of primary invasive cancer in 26 disease areas based on the relevant *International Classification of Diseases for Oncology*, *3*^*rd*^ *Edition* primary site and histological type codes routinely available in USCS data [9]. Disease areas include the following: brain cancer, breast cancer overall, human epidermal growth factor receptor 2 (HER2)–positive breast cancer, triple-negative breast cancer (TNBC), hormone receptor–positive HER2− breast cancer, cervical cancer, colorectal cancer, endometrial cancer, esophageal cancer, acute myeloid leukemia, chronic lymphocytic leukemia, liver cancer, non-small cell lung cancer, small cell lung cancer, diffuse large B-cell lymphoma, follicular lymphoma, mantle cell lymphoma, non-Hodgkin lymphoma, melanoma, multiple myeloma, ovarian cancer, pancreatic cancer (adenocarcinoma and ductal carcinoma histologies), prostate cancer, renal cell carcinoma, squamous cell carcinoma of the head and neck, and thyroid cancer.

### Data items

Cancer registries collect detailed information on the races of each patient, including data fields for five different races for multiracial individuals. However, in publicly released data, these race categories are collapsed into a single race variable, known as the primary race of the individual. Additionally, racial subgroups are consolidated into four major categories based on population denominators available in death certificates. Primary race was therefore categorized as White, Black, Asian or Pacific Islander, American Indian/Alaska Native, and unknown or other unspecified [10].

We categorized age at diagnosis as pediatric (<15 years), adolescent and young adult (15–39 years), middle-aged (40–64 years), and senior (≥65 years). We categorized sex as male and female. We categorized ethnicity as non-Hispanic and Hispanic. Stage was classified using a merged variable that spans the time periods when three different staging schemes were used: SEER summary stage 2000, derived summary stage, and summary stage 2018. The staging criteria characterized cancers as localized, regional, distant, or unknown stage. Localized cancer is confined to the primary site, regional cancer has spread directly beyond the primary site (regional extension) or to regional lymph nodes, and distant cancer has spread to other organs (distant extension) or remote lymph nodes [11].

Population estimates for rate denominators were modified annual county population estimates by age at diganosis, sex, bridged race, and ethnicity produced by the US Census Bureau in collaboration with the CDC and with support from the NCI [12]. Modifications incorporated bridged, single-race estimates that were derived from multiple-race categories in the census and accounted for known issues in certain counties. The modified county-level population estimates, summed to the state and national levels, were used as denominators in rate calculations [12].

### Data analysis

Average 5-year stabilized rates were used to ensure that annual fluctuations in the demographic distributions examined did not result in imprecise estimates of distributions for the US. Average annual rates for 2015–2019 per 100,000 population were age adjusted (using 19 age groups) to the 2000 US standard population by the direct method [13]. Corresponding 95% CIs were calculated as modified gamma intervals [14]. To determine differences between subgroups, rate ratios were calculated [15]. Rates were calculated using SEER*Stat software version 8.4.2 [16].

For all data queries, we conducted robustness tests by year of diagnosis to ensure that the relevant incidence rate ratios did not vary substantially across years by a nominal 10%, as an index of the stability of the distributions examined. We additionally derived annual US incidence estimates by dividing the 5-year stabilized estimates from USCS by 5. We then created a calculator functionality in Microsoft Excel that allows the user to input the total clinical study sample size and obtain calculated information on the representative study sample size based on the distribution from the USCS data (S1 File). Case counts of <50 were indicated as having a small sample size to emphasize the importance of representativeness beyond the relative distribution of disease in the general population.

## Results

Results for the AIR Calculator and SEER*Stat data query bank are available in S1 File. The spreadsheet may be used to calculate representative distributions given the sponsor-designated study size. An indicator of small sample size for calculated subgroups of <50 is used to encourage the sponsor to consider factors in addition to disease distribution in the US when determining whether a certain number of participants is needed for meaningful interpretation of the effect of the medicine or device on safety and efficacy in a US population. The data query bank of simulation settings files allows users to customize the selection criteria and check resulting differences in SEER*Stat.

An example excerpt of the AIR Calculator’s output in the TNBC disease area (all stages by race and ethnicity) is provided in the Table 1.

**Table 1:** Example of AIR calculator output categorized by race and/or ethnicity in early TNBC disease area [8].

Representative trial sample sizes (one-year)				5-year stabilized data: counts and age-adjusted incidence rates of women in the US diagnosed with early TNBC from 2015 to 2019, by race and ethnicity									
Race/ ethnicity	Annual US incidence (N)	%	Trial sample size (n)	Count	%	Rate	LCI	UCI	Rate Ratio	LCI	UCI	Ratio	Population
												*P*-Value	
**eBC** ^a^													
**By race/ ethnicity**													
NH White	15,795	64.5%	645	78,975	64.5%	11.8	11.7	11.9	1.00	Reference			508,234,510
NH Black	5,158	21.1%	211	25,789	21.1%	22.5^b^	22.2	22.8	1.91	1.88	1.93	<0.001	110,548,817
Hispanic (All Races)	2,363	9.7%	97	11,817	9.7%	10.0^b^	9.8	10.2	0.85	0.83	0.86	<0.001	143,504,316
NH Asian or Pacific Islander	915	3.7%	37	4,576	3.7%	8.2^b^	7.9	8.4	0.69	0.67	0.71	<0.001	52,100,572
NH American Indian/Alaska Native	137	0.6%	6	687	0.6%	9.9^b^	9.2	10.7	0.84	0.78	0.91	<0.001	6,902,258
NH Unknown or other unspecified	114	0.5%	5	568	0.5%	^c^	0						
**By race**				122,412	100.0%								
White	17,926	73.2%	732	89,629	73.2%	11.6	11.5	11.6	1.00	Reference			636,049,383
Black	5,272	21.5%	215	26,361	21.5%	21.9^b^	21.6	22.1	1.89	1.86	1.92	<0.001	118,829,712
Asian or Pacific Islander	937	3.8%	38	4,683	3.8%	8.1^b^	7.9	8.4	0.7	0.68	0.72	<0.001	54,708,235
American Indian/Alaska Native	145	0.6%	6	724	0.6%	7.0^b^	6.5	7.6	0.61	0.56	0.65	<0.001	11,703,143
Unknown or other unspecified	203	0.8%	8	1,015	0.8%	^c^	0						
**By ethnicity**				122,412	100.0%								
Non-Spanish-Hispanic-Latino	22,119	90.3%	903	110,595	90.3%	13.1	13	13.2	1.00	Reference			677,786,157
Spanish-Hispanic-Latino	2,363	9.7%	97	11,817	9.7%	10.0^b^	9.8	10.2	0.76	0.75	0.78	<0.001	143,504,316
**Total**	24,482	100%	1,000	122,412	100%								

CI, confidence interval; eBC, early breast cancer; LCI, lower CI; NH, non-Hispanic; TNBC, triple-negative breast cancer; UCI, upper CI; US, United States.

^a^ Localized/ regional based on merged summary stage variable developed by the National Cancer Institute (https://seer.cancer.gov/tools/ssm/SSM2018-BREAST.pdf)

^b^ The rate ratio indicates that the rate is significantly different than the rate of the Reference (*P*<0.05).

^c^ Statistic could not be calculated.

Note: Rates are per 100,000 and age-adjusted to the 2000 US Std Population (19 age groups—Census P25-1130) standard; Confidence intervals (Tiwari mod) are 95% for rates and ratios.

An example summary of the 5-year stabilized estimates on the right may be embedded as a separate table in a diversity action plan [1] and described as follows:

Of the 122,412 women diagnosed with early TNBC between 2015 and 2019, 64.5% were non-Hispanic White women, 21.1% were non-Hispanic Black women, 9.7% were Hispanic, 3.7% were non-Hispanic Asian or Pacific Islander, and 0.6% were non-Hispanic American Indian/Alaska Native. Five-year stabilized counts, population denominators, and age-adjusted incidence rates (AAIRS) of women in the US diagnosed with early TNBC between 2015 and 2019 and organized by race/ethnicity are presented in the Table. AAIRs varied by race/ethnicity during this time period (per 100,000): 11.8 for non-Hispanic White women; 22.5 for non-Hispanic Black women; 10.0 for Hispanic women; 8.2 for non-Hispanic Asian or Pacific Islander women; and 9.9 for non-Hispanic American Indian/Alaska Native women. Relative to non-Hispanic White women, this corresponded to an approximate 91% higher incidence rate for non-Hispanic Black women (incidence rate ratio [IRR] = 1.91; 95% CI = 1.88–1.93; *P*<0.001), 15% lower incidence rate for Hispanic women (IRR = 0.85; 95% CI = 0.83–0.86; *P*<0.001), 31% lower incidence rate for non-Hispanic Asian or Pacific Islander women (IRR = 0.69; 95% CI = 0.67–0.71; *P*<0.001), and 16% lower incidence rate for non-Hispanic American Indian/Alaska Native women (IRR = 0.84; 95% CI = 0.78–0.91; *P*<0.001). This example is for a clinical study with a target sample size of 1000 participant.

An example description of the representative trial sample size numbers on the left could mention that the representative trial sample size distribution was obtained by dividing the 5-year annual incidence frequencies by 5 to obtain annual US incidence numbers by race/ethnicity and multiplying the race/ethnicity-specific percentages by the total trial size to obtain the representative trial sample size numbers in the Table. Importantly, enrollment of a small sample size of patients in any given population may not be representative of the US, even if the projected enrollment percentages are similar to the US demographic data. Therefore, the study sponsor may need to consider enrolling sufficient numbers to allow for meaningful interpretation of the effect of the medical product on the safety and efficacy in a US population.

## Discussion

The AIR Calculator and SEER*Stat data query bank provide information on the US distribution for 26 oncology disease areas by age at diagnosis, sex at birth, and race and ethnicity, stratified by stage at diagnosis. These data may be used to understand the distribution of disease in the US by demographic characteristics. The calculator functionality allows the user to input the total clinical study sample size and obtain calculated information on the representative sample size based on the distribution from the USCS database. The data items in the AIR Calculator (ie, age at diagnosis, sex, and race and ethnicity) may be categorized differently than those in each clinical study. Additionally, clinical study teams may have inclusion or exclusion criteria that may need customization. We provide simulation settings files for users to customize and refine the categorization and case definitions and to conduct additional robustness checks as needed.

Due to lag times for data quality and completeness for cancer registry operations, the most recent year of cancer registry currently available are through 2021. The COVID-19 pandemic disrupted access to routine cancer care in 2020, which impacted the interpretation of USCS data [17, 18]. Incidence data in the second year following the pandemic remained low for some cancer types [19]. In order to capture accurate and recent data, we restricted our study to include the 5-year span from 2015–2019. This provided a 5-year stabilized estimate of the US cancer population by age at diagnosis, sex, and race and ethnicity, at a time when routine cancer care was also available.

Strengths of our study include its use of comprehensive data covering the entire US population with interstate agreements in place to exchange data and avoid duplication, focus on diverse patient populations, provision of an open resource, and commitment to transparency. These strengths contribute to the study’s reliability and potential impact on improving diversity and inclusion in clinical studies. The AIR Calculator is subject to known limitations of cancer registry data and may not have detailed information relevant to eligibility criteria for specific clinical studies at a study level. Cancer registries collect information on incident tumors and survival outcomes but not on recurrence outcomes or patterns of care. Furthermore, cancer registries collect information on the first course of treatment only and do not collect information on additional lines of therapy, or detailed information on patterns of care. Certain clinical study eligibility criteria such as detailed medical history are not routinely collected by cancer registry data, and hence, cannot be included in the AIR Calculator. Moreover, the AIR Calculator does not provide information at a study level and may not always be an appropriate method to ensure that clinical study populations are representative of the patients who may use the medical product if approved. Results from the AIR Calculator should be compared to those from a real-world data (RWD) assessment at a study level to examine potentially substantive differences in distributions between the indication level (AIR Calculator) and the study level (RWD assessment).

Additionally, data quality and completeness are subject to variations in data collection and reporting practices across different states and regions covered by population-based cancer registries. Importantly, the validity of registry-reported race and ethnicity is not well understood [20–24]; hospital policy and practice differ regarding the collection of data [25]. Nonetheless, USCS data remains a valuable resource and the most complete database available for information on a representative distribution of cancers in the US. The USCS database includes data from a wider range of cancer registries and is considered less biased in terms of race and ethnicity than SEER, which selectively oversamples specific racial and ethnic subgroups. For example, for HER2+ early breast cancer, SEER17 data make up approximately 26% of the overall tumor volume data in the USCS database and include approximately 22% non-Hispanic White patients, 24% non-Hispanic Black patients, 39% Hispanic patients, 54% non-Hispanic Asian or Pacific Islander patients, and 28% non-Hispanic American Indian/Alaska Native patients. Moreover, SEER data substantively overrepresent Hispanic and non-Hispanic Asian or Pacific Islander patients with HER2+ early breast cancer.

An important consideration is that public use of cancer registry data do not account for multiracial individuals and do not align with current standards for federal data collection on race and ethnicity by the Office of Management and Budget [26]. Specifically, because these data standards were not implemented in death certificates in all states until 2017, it is not possible to disaggregate the Asian and Native Hawaiian and Other Pacific Islander populations [27]. For epidemiological assessments informed by USCS data to align with current Office of Management and Budget standards, publicly released data would need to include a separate category for individuals identifying as multiracial and disaggregate the Asian and Native Hawaiian or other Pacific Islander populations. Additionally, our study is limited by the exclusion of Puerto Rico data from the US Cancer Statistics database. Another limitation of our study is that the AIR Calculator can only be applied to US trials or the US portion of a global clinical trial. Additional resources need to be utilized and/or developed to understand the disease burden in other countries and ensure those populations are adequately represented in clinical studies.

## Conclusions

The AIR Calculator may be a resource for clinical study planning purposes. However, results should be complemented with data from other sources to provide a more comprehensive understanding of the distribution of disease at the study level. More comprehensive data collection and alignment on data sources within industry are necessary to ensure that benchmarks in historically underrepresented patient populations are consistent, accurate, and transparent. While the AIR Calculator serves as a valuable resource for supporting diversity action plans, it is important to recognize that its results should be supplemented with data from other sources.

## Supporting information

S1 File Results for the AIR calculator and SEER*stat data query bank. (ZIP)
