# Correction: Advancing inclusive research calculator for oncology disease areas: A resource to support the development of enrollment targets in diversity action plans for industry sponsors

**DOI:** 10.1371/journal.pone.0324115

**Published:** 2025-05-12

**Authors:** David J. Press, Spencer L. James, Bruno Jolain, Nicole Richie

S2 File is omitted from the list of Supporting Information. It can be viewed below.

## Supporting information

S2 File Advancing Inclusive Research (AIR) calculator. (XLSX)
